# The Impact of Cone-Beam Computed Tomography Exposure Parameters on Peri-Implant Artifacts: A Literature Review

**DOI:** 10.7759/cureus.23035

**Published:** 2022-03-10

**Authors:** Paweł Sawicki, Paweł J Zawadzki, Piotr Regulski

**Affiliations:** 1 Department of Cranio-Maxillofacial Surgery, Oral Surgery, and Implantology, Medical University of Warsaw, Warsaw, POL; 2 Department of Dental and Maxillofacial Radiology, Medical University of Warsaw, Warsaw, POL; 3 Center of Digital Science and Technology, Cardinal Stefan Wyszyński University in Warsaw, Warsaw, POL

**Keywords:** radiation protection, dental implants, radiation, metal artifacts, cbct

## Abstract

Cone-beam computed tomography (CBCT) amounts to an excellent diagnostic tool to evaluate the peri-implant bone thickness in all dimensions. Despite the unquestionable advantages of CBCT, potential artifacts around dental implants might disturb the proper assessment of the surrounding structures. The artifacts may mask osseointegration, shallow bone defects, and other types of radiolucency, which make it difficult to establish an early diagnosis of bone loss. Proper diagnosis of bone defect is necessary to decide about surgical intervention. The aim of this literature review is to assess the CBCT exposure causing artifacts on the peri-implant structures. An electronic search of MEDLINE (PubMed) database includes studies published before July 2021 and supplemented by manual research. Clinical, ex vivo, in vitro, and animal studies evaluating the relationship between exposition parameters and occurrence of artifacts around the dental implant in CBCT studies were included. A literature review revealed that kilovoltage, tube current, and field of view may affect the occurrence of artifacts around dental implants, all of which would compromise radiological evaluation. Therefore, it is feasible to reduce the incidence of artifacts and improve the image quality by appropriate modification of the exposure parameters. However, the reduction of artifacts is often associated with a significant increase in radiation exposure; hence, an effort should be made to minimize the radiation dose in line with the ALARA (as low as reasonably achievable) principle.

## Introduction and background

Nowadays, dental implants are the best and increasingly popular method for replacing missing teeth. Planning implant treatment requires detailed diagnosis, especially in terms of bone quantity and quality. Panoramic and periapical radiography is used as a preoperative diagnostic radiological method. Its constraints, such as overlapping structures and the lack of possibility to assess bone volume, limit the preimplantation treatment planning. Nowadays, cone-beam computed tomography (CBCT) allows for a three-dimensional assessment of the quality and volume of bone tissue at reasonable cost and dose and remains the gold standard [1]. In modern CBCT devices, with an appropriate setting of exposure parameters, the effective dose is relatively small and expected to be 70 µSv for a field of view of 8 cm x 8 cm and 121 µSv for a field of view of 15 cm x 15 cm [2].

Patients after implant placement are at the risk of peri-implantitis, which might, in turn, lead to the loss of the dental implant. A systematic literature review among patients with fixed partial dentures showed that the prevalence of peri-implantitis was 9.6% in the general population and 14.3% among patients with periodontal disease [3]. Peri-implantitis is usually a chronic condition, which might lead to advanced bone loss, especially in the absence of regular, postoperative follow-ups. Effective diagnosis is of great importance when considering surgical intervention. Despite the advantages of CBCT, the authors point to the masking of the osseointegration, shallow bone defects, and other types of radiolucency, which makes it difficult to establish an early diagnosis of bone loss. This is caused by the presence of artifacts, i.e., parts of the image that do not represent any anatomical structure within the subject being evaluated and might be most commonly generated around the radiodense elements in a radiographic image [1].

CBCT images might contain various types of artifacts such as beam hardening phenomenon, photon starvation, scatter, partial volume effect, undersampling, exomass, detector miscalibration, and patient motion [4]. Exposure of objects containing metals from which dental implants are made is associated with the formation of artifacts that reduce image quality. They might disturb proper assessment of the surrounding structures [5].

**Figure 1:**
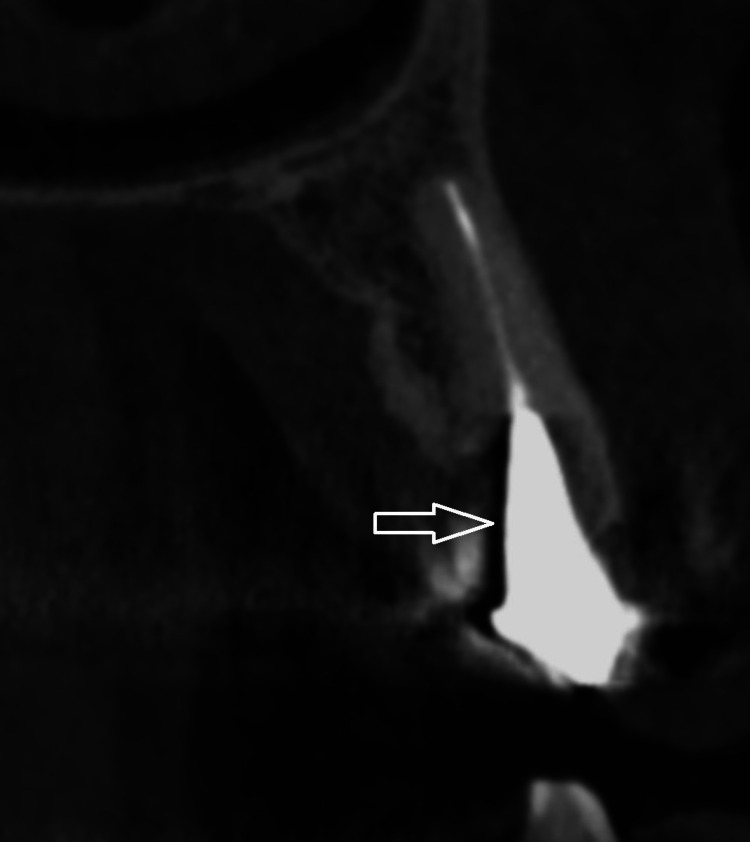
Beam hardening artifacts adjacent to metal post and core in anterior maxillary tooth Image credit: The authors of the current study.

**Figure 2:**
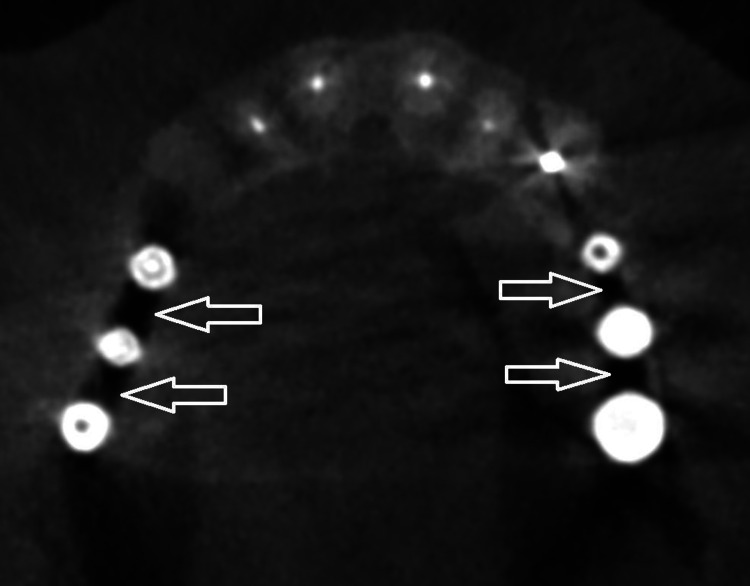
Dark streaks between dental implants in cone-beam computed tomography (type of beam hardening artifacts) Image credit: The authors of the current study.

Beam hardening phenomenon and photon starvation are the main causes of peri-implant artifacts. Beam hardening occurs when a polychromatic x-ray beam passes through an object, resulting in selective absorption of lower energy (lower wavelength) photons and thus increased beam energy [6]. The higher the density and the atomic number of an object, the higher the number of absorbed photons [7]. The mechanism underlying CBCT artifacts is the same as the one in computed tomography (CT). However, artifacts are more prominent in CBCT compared to CT due to the lower tube voltage [5]. Two types of artifacts are generated as a result of beam hardening: cupping artifacts, caused by a non-linear x-ray beam attenuation and dark bands or streaks between highly dense objects (Figures 1, 2) [8].

**Figure 3:**
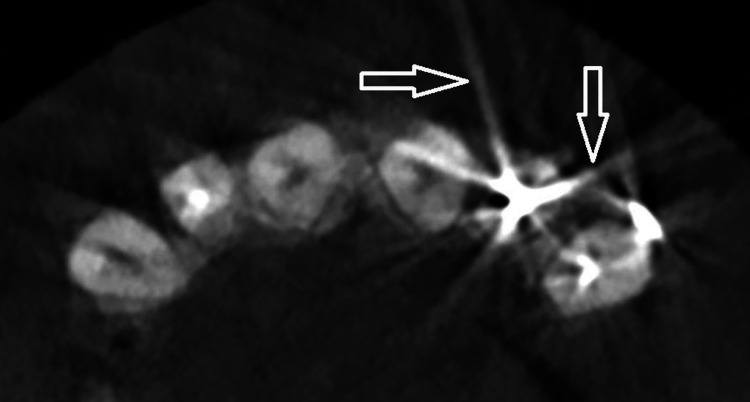
Photon starvation effect generates a large amount of noise and streaks around metal post and core in the anterior maxillary tooth Image credit: The authors of the current study.

Photon starvation in CBCT often occurs around prosthetic crowns and implants. When the x-ray beam is traveling horizontally, the attenuation is greatest, e.g., when it passes through very dense objects. This generates a large amount of noise and streak artifacts around highly saturated objects (Figure 3) [9]. Effects similar to the ones seen for beam hardening and photon starvation are caused by scattering.

The main objective of this article is to provide an in-depth discussion on the impact of CBCT exposure parameters on peri-implant assessment feasibility taking into consideration the available literature. In the vast majority of CBCT machines, all exposure parameters can be set manually within a given range. This not only allows to change the dose of absorbed radiation but also influences the incidence of artifacts in the field of view (FOV). In clinical practice, the quality of CBCT images depends on multiple factors, such as machine model, FOV, type of object scanned, exposure time, x-ray tube voltage, and tube current as well as spatial resolution defined by the size of the imaging voxels [10]. Furthermore, manufacturers offer a metal artifact reduction tool (MAR) based on a reconstruction algorithm [11]. MAR software activation reduces the interference caused by metals and may improve the image quality [12].

## Review

Search strategy and selection criteria

The main objective of this article is to analyze the impact of designated CBCT exposure parameters on the inherent peri-implant artifacts taking into consideration the available literature. The MEDLINE (PubMed) bibliographic database was searched for studies published before July 2021 and supplemented by manual research. The search strategy was restricted to English language publications using the following combined terms: (dental OR dentistry) AND implant AND (artifacts OR artifacts).

Studies and reviews evaluating the relationship between exposition parameters and occurrence of artifacts around the dental implant in CBCT studies were included. Titles and abstracts were screened based on the inclusion criteria. Case reports were not included. Publications not fulfilling the eligibility criteria were not included in this analysis. During the procedure, studies for which full texts could not be obtained were excluded. The full text of the selected papers was reviewed, and the relevant data on the impact of exposure parameters on peri-implant artifacts were extracted.

**Figure 4:**
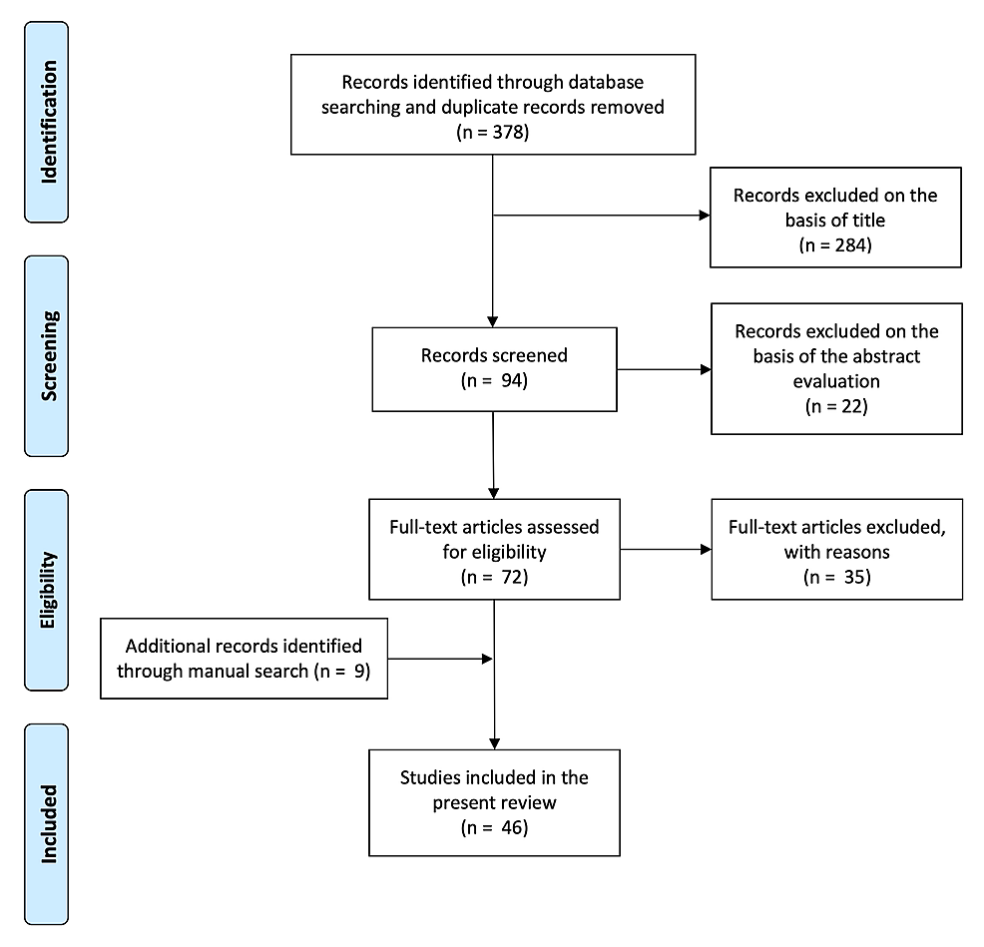
PRISMA diagram of the included studies The image shows the selection process for the studies included according to the PRISMA statement. PRISMA: Preferred Reporting Items for Systematic Reviews and Meta-analyses.

After removing duplicates, references were screened, and 378 titles were found and considered eligible for further consideration. A total of 284 titles were excluded based on title evaluation. Initial review of the abstracts resulted in 72 articles that were considered for full-text review. A total of 35 papers had to be excluded at this stage because they did not fulfill the inclusion criteria. Nine additional records identified through the manual search were included. Forty-six articles were included in the present review. Figure 4 shows a detailed flowchart of the literature review search and selection process according to the Preferred Reporting Items for Systematic Reviews and Meta-analyses (PRISMA) statement.

X-ray tube voltage and tube current

Low x-ray beam energy, high density of materials, and a high atomic number of the scanned object contribute to beam hardening [7,13]. Increasing the x-ray tube voltage increases the beam energy and improves x-ray penetration, resulting in image contrast and noise reduction as well as in the irradiation dose increment [5,13,14]. This leads to the reduction of the overall number of beam hardening artifacts (increased contrast-to-noise ratio [CNR]) [11,13]. However, the number of scatter artifacts caused by the photons with different energies increases in the low-voltage images [15]. Therefore, it is advisable to set the exposure parameters in a compromising manner to get an optimal reduction of both beam hardening and scatter. Beam hardening artifacts and scatter are present in each CBCT image but with a different intensity depending on x-ray voltage. A high x-ray value (e.g., 90 kV) allows to reduce the following artifacts but increases the radiation dose. Therefore, it is difficult to determine the ideal value of x-ray tube voltage. The authors of this article conducted studies aiming to determine the ideal x-ray tube voltage for postoperative implant therapy follow-up. Panjnoush et al. showed that a change in the tube voltage from 70 kVp to 84 kVp had no effect on the presence of artifacts in the spaces between metallic objects in dental applications [16]. It was shown that the incidence of artifacts for zirconium implants is higher compared to that of titanium implants. The reason for higher artifacts occurrence with zirconium implants is the difference in the atomic number of zirconium (Zr, atomic number = 40) and titanium (Ti, atomic number = 22), as shown in the previous part of the article [11].

**Figure 5:**
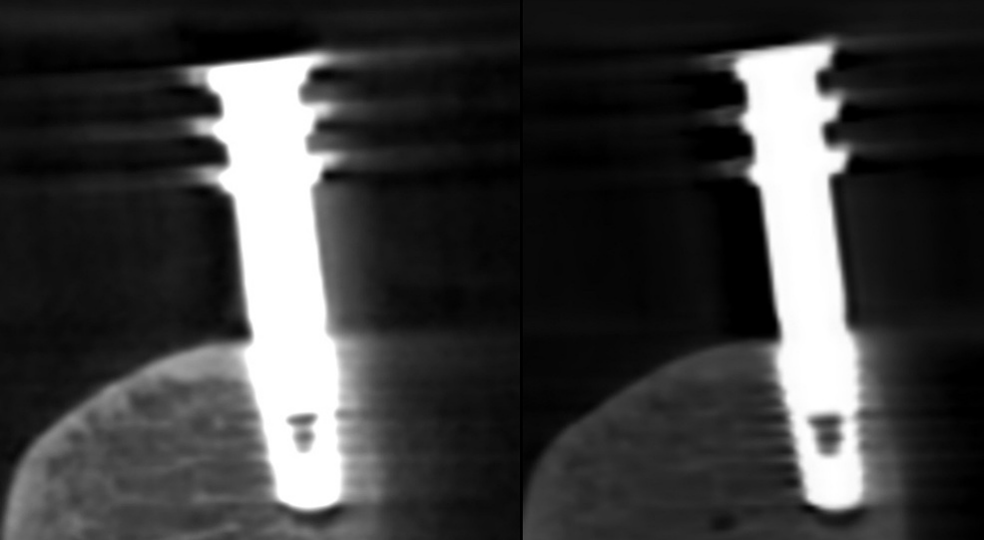
Bone model with a dental implant with impression transfer attached exposed to a tube voltage of 60 kV (left) and 90 kV (right) The remaining exposure parameters are constant. Lower voltage limits the diagnosis of peri-implant buccal plate defects. Image credit: The authors of the current study.

The difference in the number of artifacts generated as a result of changes in the tube voltage is shown in Figure 5. A bone model with a dental implant was exposed to tube voltages of 60 kV and 90 kV with constant other exposure parameters. Exposure to 60 kV significantly limited the diagnosis of peri-implant buccal plate defects.

The cathode tube current has a significant influence on the radiation dose during CBCT exposure. An increase in mAs (the product of the lamp tube current and the exposure time) reduces the noise but increases the radiation dose [14]. Studies showed no effect of changes in the tube current (within the range available in the CBCT) on the formation of peri-implant artifacts [16-18]. In another study, Fontenele et al. found that a change in the tube current and metal artifact reduction algorithms (MAR) did not affect the diagnostic efficacy for vertical root fractures in endodontically treated teeth adjacent to zirconium implants [19].

Field of view

Increasing the FOV exposes more tissues to x-rays, increases scattered radiation to the surrounding tissues, decreases contrast, and increases noise and radiation dose [14,20,21]. Scatter might be reduced by reducing FOV as well as the use of an anti-scatter grid and algorithms to correct the x-ray scatter [22]. Pauwels et al., who investigated the relationship between FOV and the range of rotation and an effective dose of radiation, showed that the effective dose ranged from 54 μSv for 4 cm x 4 cm to 303 μSv for 17 cm x 12 cm when using Accuitomo 170 3D CBCT system (J. Morita Corporation, Japan) [20]. It was shown that artifacts are less prominent in a small FOV, which might be due to the fact that by an increase in FOV, the irradiated area increases in size, and consequently, scattered radiation, noise, and image artifact increase. Similar results were provided by Parsa et al. [17,23]. Nikbin et al. assessed the effect of an object position in the FOV and the use of the MAR algorithm on CBCT diagnostic efficacy in vertical root fractures. Diagnostic accuracy was higher with central positioning compared to peripheral positioning, irrespective of MAR [24].

It is recommended in clinical practice to limit the FOV, if possible, to avoid scanning areas susceptible to beam hardening (such as metal restorations and implants). If the target volume is too large, it might limit the diagnostic possibilities in the region of interest (ROI) due to the overlapping of artifacts outside this region. This can be achieved by collimating the beam, changing the patient's position, and separating the dental arches during CBCT scanning [25].

CBCT rotation range

The range of tube-detector axis rotation varies depending on the CBCT system and can be changed from 180 to 360 degrees in some systems [20]. CBCT scanning with 360-degree rotation compared to the standard 180-degree scan enhances the image quality by creating more basis images. Enhancement of rotation range increases the CNR, but despite the larger amount of data, changing the device rotation range does not affect the number of artifacts (e.g., beam hardening, scatter, and ring artifacts) that occur both in 180- and 360-degree scans [7,21,26].

Increasing the range of rotation extends the exposure time, which largely determines the effective dose of x-rays. The recommendations of radiological protection in CBCT, International Commission on Radiological Protection (ICRP) Publication 129, suggest a rotation of 180° plus beam angle rotation as sufficient for tomographic reconstruction. Radiosensitive organs should be on the detector side to reduce the risk of overexposure. A full, 360-degree rotation makes it impossible to avoid exposure to these structures [27].

Voxel size

A voxel is the smallest isotropic element of a CBCT image. CBCT devices allow for setting a voxel size from a minimum of 70 to 300 microns. The voxel size determines how small objects can be differentiated and how detailed diagnosis of maxillofacial anatomical structures can be performed [28]. The partial volume effect is an artifact associated with the voxel size. It occurs when two structures are projected on the same voxel producing an intensity value being an average value of both structures [29]. In consequence, these structures cannot be differentiated and the signal-to-noise ratio (SNR) for each voxel is reduced.

Research findings on the effect of voxel size on implant assessment are contradictory. Vasconcelos et al. assessed the differences in generating artifacts using zirconium and titanium implants placed in human mandibles (ex vivo) and different tube voltage and voxel size (high resolution, 0.16 mm; low resolution, 0.32 mm) [11]. It was shown that a reduced voxel size did not affect beam hardening and scatter artifact generation [13]. In the in vitro study using an I-CAT 3D Imaging System (Imaging Sciences International, Hatfield, PA), Kursun-Cakmak et al. assessed the image quality with different voxel sizes (0.2, 0.25, 0.3, and 0.4 mm) and recommended follow-up CBCT using low-resolution settings (0.3 and 0.4 mm) due to the highest CNR. The author also found that among the implant materials tested (zirconium (Zr), titanium Grade 4 [Ti], and titanium-zirconium [Ti-ZrO_2_] alloy), titanium Grade 5 (titanium-aluminum-vanadium alloy) had the lowest impact on artifact creation, while Zr had the highest impact [30]. Bechara et al. found that the CNR value does not depend only on the voxel size but might vary for different CBCT systems. They found that a smaller voxel size does not guarantee an improved quality [26]. To summarize the following research results, it can be concluded that although reducing the size of the voxel increases spatial resolution, it also causes a deterioration of the image quality around dental implants and an unjustified increase in the radiation dose [1,14,18,26,30-32].

Software methods used to reduce artifacts

There are some software solutions that allow for the reduction of artifacts present in the acquired image, which improves its quality. Activation of the MAR algorithm, i.e., iterative reconstruction methods that allow reducing image interference is caused by metals or high-density objects by increasing the CNR [11,31,33]. The efficacy of MAR depends on the manufacturer of the radiographic machine [34]. In one study, cylinders made of titanium (Ti) and chromocobalt alloy (CrCo) were exposed to two CBCT devices: Picasso Trio (Vatech, South Korea) and ProMax (Planmeca, Helsinki, Finland). It was shown that the use of MAR for Ti cylinders significantly reduced the voxel’s mean (p ≤ .05) in the Picasso Trio CBCT machine and significantly increased the voxel’s mean (p ≤ .05) in the ProMax CBCT machine. No efficacy of MAR was demonstrated for CrCo cylinders using both machines [35]. Statistically significant (p ≤ 0.0001) efficacy of MAR in reducing amalgam, copper-aluminum alloy, and titanium artifacts was found for various CBCT machines. The relationship between the atomic number of the metal and the increased number of artifacts was confirmed. It was found that a higher metal atomic number caused greater artifact expression [36].

Nascimento et al. investigated differences in the efficacy of MAR in three tested conditions: "without MAR," with "MAR activated after the acquisition," and with "MAR activated before the acquisition." For this purpose, a zirconium oxide implant was placed in the human mandibular bone at the position of the missing lower right first molar, and exposition was performed with the OP300 Maxio system (Instrumentarium Dental Inc., Tuusula, Finland). It was shown that the cortical lingual plate had lower CNR and voxel value in the control and the implant group (p < 0.05). It was demonstrated that MAR efficacy increased with an increasing number of artifacts. No relationship was found between MAR efficacy and its activation mode [4]. It was shown that the availability of more data (images from different projections also called basis images) increases the effectiveness of MAR. The relationship between the higher number of basis images and reduced generation of artifacts in the absence of activated MAR algorithms was not shown [37,38].

Kamburoglu et al. assessed the efficacy of MAR algorithms in the diagnosis of buccal peri-implant defects. To this end, buccal peri-implant defects were performed in implants placed into human cadaver mandibles and then investigated with CBCT in artifact reduction mode at four different levels of intensity, including the non-activated mode. It was shown that there is a statistically significant influence of MAR (regardless of its mode) on the diagnosis of simulated bone loss in the applied methodology. There was a higher interobserver agreement for periodontal defects (kappa value from 0.189 to 1.000) vs. peri-implant defects (kappa value from 0.140 to 0.792). It was also found that buccal peri-implant defects are more diagnostically challenging than buccal periodontal ones. The main cause of these is the presence of artifacts related to the metal around titanium dental implants, which does not occur in periodontal assessment because of the lack of metal in tooth structures despite the teeth being after endodontic treatment and treatment with metallic post and core restorations [39]. A similar study by de-Azevedo-Vaz et al. showed no improvement in the efficacy of diagnostic assessment of peri-implant fenestration and dehiscence using MAR [40]. In their study, Bechara et al. used ProMax and Master 3D (Vatech, Hwaseong, Republic of Korea) systems to assess the efficacy of artifact reduction algorithms in the diagnosis of root fractures in endodontically treated teeth. Both machines showed higher diagnostic sensitivity and specificity for vertical root fractures when using no AR [41].

Discussion

The foregoing article sums up for the first time the influence of all the variable parameters of CBCT device exposition in the presence of artifacts around dental implants. To date, the influence of the changing exposure parameters on the possibility of evaluation of the buccal bone around dental implants has not been determined. The authors of this document are conducting studies aiming to clarify this dependency, which will have a significant impact on the possibility of regular inspection for the implant and prosthetic treatment patients. The goal of the research is the discovery of the optimal image quality settings for the exposure parameters while maintaining the lowest dose of radiation possible.

Peri-implantitis and its predictable diagnostics in CBCT images are the main areas of interest in implantology. Peri-implantitis causes gradual destruction of osseous tissue around dental implants, which can lead to loss of implants. Appropriate postoperative care and diagnostics, both clinical as well as radiological, allow for assessment of peri-implant tissues and possible implementation of treatment during the early stages of the disease. Based on the results presented above, modifications to the exposure parameters of CBCT examinations performed during postoperative follow-up are recommended, which will allow for reduction of artifacts (mainly beam hardening effects) that prevent the assessment of osseous tissue around dental implants.

The x-ray tube voltage has a major influence on the occurrence of artifacts around dental implants and metal objects in CBCT images. Increasing x-ray tube voltage decreases the occurrence of artifacts such as beam hardening and noise and increases the CNR. Increasing the tube current decreases noise but does not affect the formation of artifacts around dental implants (peri-implant artifacts). Increasing the CBCT rotation range from 180 to 360 degrees does not affect the number of artifacts, and decreasing the voxel size does not improve the image quality around dental implants. To reduce scatter artifacts, the FOV should be reduced, if possible, which will both improve the image quality and reduce radiation dose. Following modifications of exposure parameters and their impact on CBCT image and radiation dose are detailed in Table 1.

**Table 1:** Influence of exposure parameters modification on the incidence of artifacts and cone-beam computed tomography (CBCT) image quality ↑: Increase; ↓: Decrease; mAs: Tube current-exposure time product; kV: Tube voltage; MAR: Metal artifact reduction.

Exposure parameter	Artifacts (beam hardening)	Noise	Contrast-to-noise ratio (CNR)	Spatial resolution	Radiation dose
Voltage ↑	↓ [11,13]	↓ [14]	↑ [11,13]	-	↑ [14]
mAs ↑	-	↓ [14]	-	-	↑ [14]
Voxel size ↑	-	↓ [14,18,26,30,31]	-	↓ [28]	↓ [1,32]
Field of view ↑	↑ [17,23]	↑ [14,21]	↓ [14,21]	↓ [42]	↑ [14,21]
Rotation arc ↑	-	-	↑ [26]	-	↑ [27]
Metal artifact reduction	-	↓ [22,42,43]	↑ [11,31,33]	-	-

Taking into account the fact that modification of all above-mentioned parameters influences the radiation dose, only x-ray tube voltage and FOV should be modified, which are the only ones listed to influence the formation of peri-implant artifacts, leaving the other exposure parameters at settings that allow obtaining the lowest possible radiation dose. It is related to the ALARA (as low as reasonably achievable) principle proposed by the ICRP [44]. Although the principle seems commonly known, studies conducted in Turkey by Atci et al. indicate that 96% of the surveyed emergency medicine doctors and neurosurgeons of the local hospital in Istanbul did not know the meaning of the acronym ALARA, and 92% did not know the radiation doses received by their patients during brain CT [45]. As it is commonly known, the effective dose of x-ray radiation increases with increasing tube voltage. Vasconcelos et al. showed that increasing the tube voltage from 70 kVp to 90 kVp in CBCT increased the effective dose more than five-fold (4.08 µSv and 20.91 µSv at low resolution and 18.4 µSv and 93.41 µSv at high resolution, respectively) [11]. Given the significant increase in the effective dose, it should be verified before each exposure whether the benefits of better image quality outweigh the potential risks of higher radiation. The consequences of irradiation might be classified into two groups. Stochastic or linear-dose effects include cancer and hereditary changes in the offspring. The risk increases with an increase in the radiation dose. Deterministic effects involve transient or permanent tissue damage and acute radiation syndrome, which occur when cells are killed by a high dose of radiation. They are observed when doses exceeding 0.5 Gy are received, although this value might be lower for individual organs; therefore, it does not have to be taken into account in diagnostic tests such as CBCT where the absorbed dose is much lower [46].

## Conclusions

CBCT is the gold standard in the pre- and postoperative diagnosis as well as treatment of peri-implantitis. Due to the growing interest in the treatment with dental implants, many studies are currently conducted to achieve the lowest possible number of metal artifacts in CBCT. From the literature review, it might be drawn that dental artifacts are a significant limitation in the diagnosis of peri-implant tissues, and it is possible to reduce the incidence of artifacts and improve the image quality by appropriately modifying the exposure parameters. The reduction of artifacts is often associated with a significant increase in radiation; therefore, effort should be taken to minimize the radiation dose in accordance with the ALARA principle. Undoubtedly, there is a need to conduct further studies to improve the CBCT exposure protocol to improve the image quality and increase the diagnostic efficacy in peri-implant pathologies, which would allow for their early diagnosis and treatment.
